# Bone marrow suppression and severe anaemia associated with persistent *Plasmodium falciparum *infection in African children with microscopically undetectable parasitaemia

**DOI:** 10.1186/1475-2875-4-56

**Published:** 2005-12-01

**Authors:** Marie Helleberg, Bamenla Q Goka, Bartholomew D Akanmori, George Obeng-Adjei, Onike Rodriques, Jorgen AL Kurtzhals

**Affiliations:** 1 Centre for Medical Parasitology, Department of Clinical Microbiology 7602, Copenhagen University Hospital, 2100 Copenhagen, Denmark; 2 Department of Child Health, Korle-Bu Teaching Hospital, Accra, Ghana; 3 Immunology Unit, Noguchi Memorial Institute for Medical Research, Legon, Ghana

## Abstract

**Background:**

Severe anaemia can develop in the aftermath of *Plasmodium falciparum *malaria because of protracted bone marrow suppression, possibly due to residual subpatent parasites.

**Materials and methods:**

Blood was collected from patients with recent malaria and negative malaria microscopy. Detection of the *Plasmodium *antigens, lactate dehydrogenase (Optimal^®^), aldolase and histidine rich protein 2 (Now malaria^®^) were used to differentiate between patients with (1) no malaria, (2) recent cleared malaria, (3) persistent *P. falciparum *infection. Red cell distribution width (RDW), plasma levels of soluble transferrin receptor (sTfR) and erythropoietin (EPO) were measured as markers of erythropoiesis. Interleukin (IL) 10 and tumour necrosis factor (TNF)α were used as inflammation markers.

**Results:**

EPO was correlated with haemoglobin, irrespective of malaria (R = -0.36, P < 0.001). Persistent *P. falciparum *infection, but not recent malaria without residual parasites, was associated with bone marrow suppression i.e., low RDW (P < 0.001 vs. P = 0.56) and sTfR (P = 0.02 vs. P = 0.36). TNF-α and IL-10 levels were not associated with bone marrow suppression.

**Conclusion:**

In the treatment of malaria, complete eradication of parasites may prevent subsequent development of anaemia. Severely anaemic children may benefit from antimalarial treatment if antigen tests are positive, even when no parasites can be demonstrated by microscopy.

## Introduction

Anaemia is a common, life-threatening complication of *Plasmodium falciparum *malaria in African infants and young children. The anaemia develops when accelerated removal of erythrocytes is not compensated by the bone marrow. Bone marrow suppression is generally present in all malaria patients [1] and has also been described in asymptomatic *P. falciparum *infection [2]. The fact that some malaria patients develop severe anaemia, whereas others retain normal or near normal haemoglobin (Hb) must thus be explained by the amount of erythrocyte destruction during the period until return of normal bone marrow function. The anaemia may either develop rapidly with severe, acute haemolysis, or take a slow smouldering course, with a relatively slow rate of erythrocyte destruction in the presence of persistent bone marrow suppression [3]. Data on the duration of bone marrow suppression after a malaria attack are conflicting. In some studies there has been evidence of hypoproliferative erythropoiesis and dyserythropoiesis for weeks following treatment [4,5], while other studies have shown that bone marrow suppression is reversed rapidly after treatment [1,6].

In areas where malaria is endemic, a high number of children with severe anaemia but without detectable parasites are hospitalized each year coinciding with the peak of malaria transmission. The history of recent febrile illness and the presence of detectable circulating antigens strongly suggest that these children suffer from the late effects of a recent malaria attack [7]. The question is whether there are signs of bone marrow suppression in these patients, and if so, whether this bone marrow suppression is due to a sustained effect of the malaria attack or to the persistence of parasites that are undetectable by routine microscopy. In the latter case, it is possible that these patients would benefit from a repeated course of antimalarial treatment.

Patients with severe malarial anaemia have an increased ratio between the pro-inflammatory cytokine, tumour necrosis factor (TNF)-α, and the anti-inflammatory cytokine, interleukin (IL)-10 [8], and it has been proposed that inflammatory cytokines may be a causative factor for malarial anaemia [9,10]. If so, protracted bone marrow suppression in the aftermath of a malaria attack might be due to persistence of a dysregulated inflammatory response. However, mild and moderate malarial anaemia is not associated with an inverse ratio between TNF-α and IL-10 [2].

The purpose of the present study was to test the hypothesis that an impaired erythropoietic response to anaemia is associated with a persistent malarial infection in patients without microscopically detectable parasites. In order to differentiate between recent and persistent *P. falciparum *infection, two rapid tests for soluble malarial antigens, Now^® ^ICT Malaria P.f/P.v (Binax, US) and Optimal^® ^(DiaMed AG, Schweiz) were performed. Binax is based on detection of histidine rich protein 2 (HRP2) and aldolase and can remain positive for weeks following treatment of the infection [11]. Optimal^®^, on the other hand, detects parasite derived lactate dehydrogenase (pLDH) and test results are positive only in the presence of live parasites [12].

Red cell distribution width (RDW) and plasma levels of erythropoietin (EPO) and soluble transferrin receptor (sTfR) were measured as markers of erythropoiesis. RDW is a measure of size variation in red blood cells and is increased when erythropoiesis is stimulated [13]. sTfR is secreted mainly by erythroblasts, and plasma levels are increased when turnover in the bone marrow is raised as in haemolytic anaemia, whereas levels are normal in the anaemia of chronic disease [14]. Plasma levels of TNF-α and IL-10 were analysed to determine if the bone marrow suppression was correlated to dysregulated inflammation.

## Materials and methods

The study took place at the Department of Child Health, Korle Bu Teaching Hospital, Accra, Ghana during the malaria season, July and August, 2003. Two groups of children aged 0.5–12 years were consecutively recruited. Both groups consisted of children with a presumptive or confirmed diagnosis of recent malaria but without detectable parasites in Giemsa-stained blood films, examined by ordinary microscopy.

### Group 1

Patients admitted to the emergency room with a presumptive diagnosis of acute malaria but with a negative microscopic test for malaria. The majority of these patients had received treatment for malaria either at home or in various health facilities prior to being referred to hospital. Those who had haemoglobin (Hb) ≤ 5 g/dl or Hb ≥ 8 g/dl were included, unless they had a history of a recent trauma or bleeding. Four hundred and five patients with the same age and sex distribution and with proven malaria by microscopy acted as a positive control group.

### Group 2

Children who came for follow-up on day three after initiation of treatment for acute, uncomplicated falciparum malaria at the health clinic attached to the hospital and who had cleared their parasitaemia microscopically. Patients with febrile illness other than malaria, with severe malaria, concomitant infections or known chronic disease were excluded. The children had received amodiaquine, artesunate or a combination of the two as part of an ongoing drug trial.

Children were enrolled after informed consent from parents or guardians. The ethics and protocol review committee at the University of Ghana Medical School had approved the study.

Blood was collected in EDTA-coated tubes and examined using an automated haematology analyser (Sysmex, KX-21, Germany). Giemsa-stained blood films were prepared on day 0 (*group 1*) and on day 0 (i.e. prior to inclusion), 3 and 7 (*group 2*). Blood films were considered to be negative if 200 leucocytes had been counted without finding parasites, corresponding to a detection level of approximately 50 parasites/μL. Antigen detection was performed on whole blood from day 0 (*group 1*) and day 3 (*group 2*). The rapid diagnostic tests, Now^® ^ICT Malaria P.f/P.v (Binax, US) and Optimal^® ^(DiaMed AG, Schweiz), were used according to the manufacturer's instruction.

The EDTA-plasma was subsequently collected by centrifugation and stored at -20°C until analysis. Plasma concentrations of EPO, sTfR, TNF-α and IL-10 were measured using commercial ELISA kits according to the manufacturer's instructions (EPO, sTfR and IL-10, R&D Systems, MN, USA and TNF-α, BioSource International, CA, USA).

### Statistics

All data, except haemoglobin, were logarithmically transformed to achieve normal distribution. Differences between groups were analysed using two-tailed student's t-test, except differences in haemoglobin levels that were analysed by Mann Whitney two sample rank sum test. Associations between parameters were analysed by multiple regression analyses. P-values < 0.05 were considered statistically significant. All calculations were performed using Stata SE 8.0 (Stata Corporation, TX, USA)

## Results

Forty eight patients were enrolled into *group 1*: 24 with severe anaemia and 24 with Hb ≥ 8 g/dl. Two (8.3 %) of the patients with severe anaemia and 15 (62.5 %) of those with Hb ≥ 8 g/dl had no detectable malaria antigens and were, thus, unlikely to suffer from malaria ('No malaria', Table 1). Of the remaining 31 patients, all had a positive HRP-2 test. Sixteen also had a positive pLDH test, suggestive of persistent malaria, whereas 15 had a positive HRP-2 test only, suggestive of recent malaria. Among the patients with a positive antigen test, 25 reported to have taken some antimalarial medication prior to admission, four had not received treatment and information was missing for two patients. Forty seven patients were enroled into *group 2*. Their median parasite density on day 0 was 25,633 parasites/μL (25th – 75th percentiles: 5096 – 70,755). On the day of inclusion (day 3) all had undetectable parasitaemia by microscopy but had a positive HRP-2 test. Only eight had signs of persistent parasitaemia as indicated by the pLDH test. In both groups, patients with persistent malaria tended to have lower Hb than those with recent malaria; although in *group 1*, the difference did not reach statistical significance (*group1*: P = 0.19 and *group 2*: P = 0.04, Table 1). In order to simplify the result presentation, the two groups are combined, based on the assumption that subpatent *P. falciparum *infection played a similar role in both groups. However, since the groups had different age distribution and the clinical similarity between the groups could not be established, the data were also analysed separately for the groups. As indicated in the text, this did not affect the conclusions of the study.

**Table 1:** Patient characteristics

	Group 1			Group 2	
		
	Persistent malaria	Recent malaria	No malaria	Persistent malaria	Recent malaria
Number of patients	16	15	17	8	39
Age^1 ^(years)	2 (1–12)	4 (0.5–10)	2 (0.5–12)	5.8 (1.5–10)	5.8 (1–12)
Temperature on admission^1 ^(°C)	37.0 (36.0–38.5)	38.0 (36.7–38.6)	37.5 (36.6–39.5)	37.2 (36.8–38.8)	38.2 (36.0–40.0)
Sex (boys:girls)	12:4	7:8	10:7	5:3	23:15
Haemoglobin^1 ^(g/dl)	3.4 (2.1–10.2)	4.4 (1.6–11.6)	9.8 (1.5–11.8)	6.6 (4.7–10.5)	9.2 (5.2–12.0)
TNF-α (pg/ml)^2^	16.2 (6.9–38.0)	13.5 (6.5–27.5)	5.4 (1.2–23.4)	6.2 (0.6–63.1)	8.7 (5.5–13.5)
IL-10 (pg/ml)^2^	56.2 (15.8–208.2)	29.5 (7.8–112.2)	3.0 (0.9–9.8)	8.9 (3.6–21.9)	8.3 (5.8–11.7)
IL-10/TNF-α^2^	1.9 (1.0–3.6)	1.4 (0.4–5.6)	1.0 (0.5–2.3)	1.7 (0.1–46.8)	1.0 (0.5–2.0)

1: median (range)

2: mean (95% CI)

### Erythropoiesis

**Figure 1:**
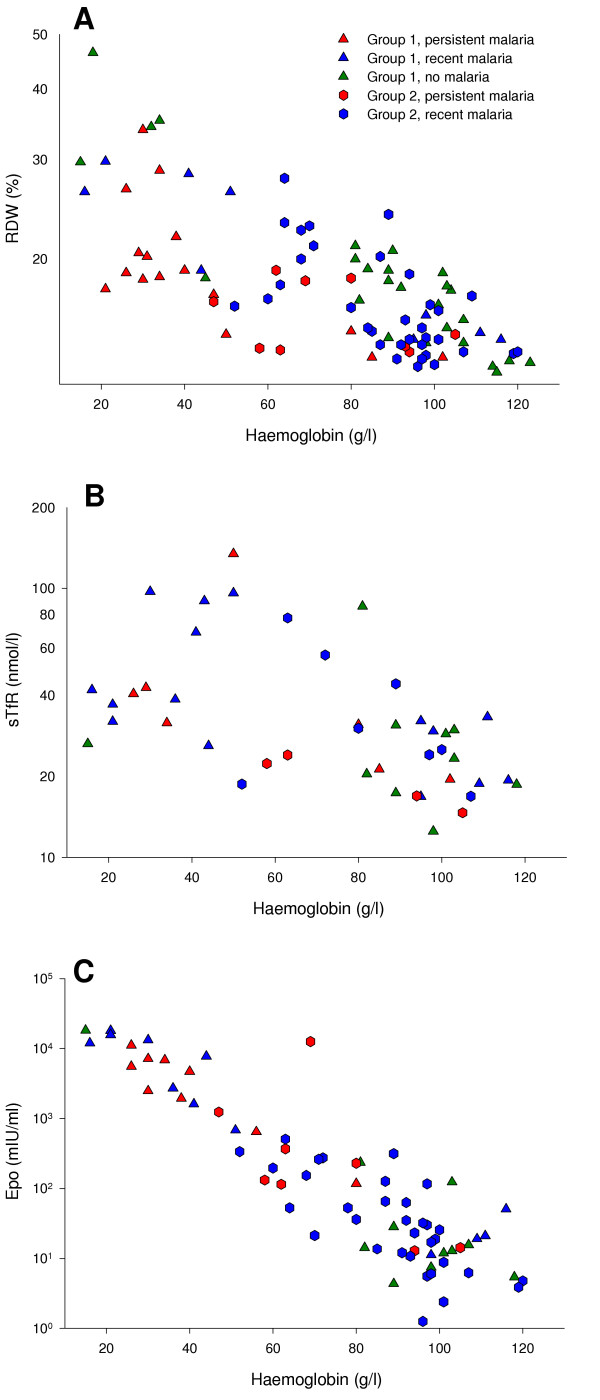
**Effect of *P. falciparum *on erythropoietic response to anaemia**. Associations between haemoglobin and (A) red cell distribution width (RDW), (B) soluble transferrin receptor (sTfR), and C. erythropoietin (EPO) in children with recent malaria (pLDH negative, HRP2 positive), persistent, submicroscopic *P. falciparum *infection (pLDH and HRP2 positive) or without signs of malaria (pLDH and HRP2 negative).

**Figure 2:**
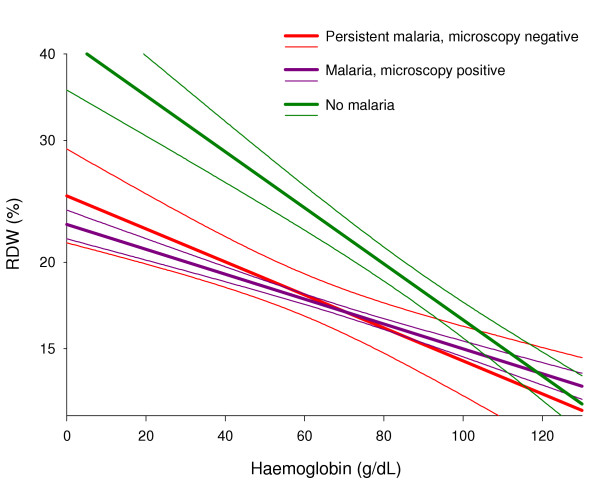
**Comparison of the effect of patent versus subpatent *P. falciparum *infection on erythropoietic response to anaemia**. Association between haemoglobin and red cell distribution width (RDW) in microscopy negative children with persistent malaria, children with microscopically detectable parasites and children without malaria. Regression lines and 95% confidence intervals.

The bone marrow response to low Hb was compared between patients with positive pLDH, (persistent malaria, n = 24) and those with negative pLDH, (recent malaria or no malaria, n = 71) in order to detect an effect of persistent *P. falciparum *infection on erythropoiesis. Both RDW and sTfR increased in response to low Hb as indicated by a negative correlation between RDW and Hb and between sTfR and Hb. However, the levels of RDW and sTfR were lower in patients with persistent infection (filled symbols) than in those with recent malaria or without signs of malaria (open symbols, Figure 1A and 1B). In a multivariate regression analysis with haemoglobin and test results for pLDH and HRP2 as explanatory variables, a positive test for pLDH, but not a positive test for HRP2 alone, was significantly associated with reduced levels of RDW and sTfR (Table 2). For this analysis, all patients in *group 1 *and *group 2 *were combined in order to span the range of Hb-levels. However, when the multiple regression analysis was repeated for the two groups separately, the results were similar, although the association between pLDH and sTfR did not reach statistical significance (log RDW: *group 1*: P < 0.001 and *group 2*: P < 0.05; log sTfR *group 1*: P = 0.07 and *group 2*: P = 0.08). Persistent, microscopy negative malaria reduced the bone marrow response to the same extent as patent malaria. Thus, the slope of the regression line between RDW as the dependent and Hb as the independent variable was reduced to the same extent in microscopy negative patients with persistent malaria as in the 405 patients with a positive microscopy compared to patients without malaria (Figure 2, red slope, purple slope, and green slope, respectively, P < 0.001 for the comparison between no malaria and both other groups).

**Table 2:** Association between markers of erythropoiesis and *Plasmodium *antigens in multiple regression analyses with haemoglobin and test results for parasite derived lactate dehydrogenase (pLDH) and histidine rich protein 2 (HRP2) as explanatory variables.

	pLDH		HRP2		Haemoglobin	
	β (95% CI)	P-value	β (95% CI)	P-value	β (95% CI)	P-value

Log RDW^1 ^(%)	-0.08 (-0.11–(-0.04))	<0.001	-0.04 (-0.12–0.04)	0.56	0.03 (0.02–0.04)	<0.001
Log sTfR^2 ^(nmol/l)	-0.18 (-0.32–(-0.03))	0.02	0.08 (-0.09–0.24)	0.36	0.04 (0.02–0.06)	<0.001
Log EPO^3 ^(mIU/ml)	0.13 (-0.16–0.43)	0.39	0.14 (-0.18–0.47)	0.38	0.34 (0.30–0.39)	<0.001

1: Red cell distribution width

2: Soluble transferrin receptor

3: Erythropoietin

Plasma levels of EPO showed a strong inverse correlation with Hb (Figure 1C, R = -0.36, 95%CI: -0.40 – -0.32, P < 0.001), and neither pLDH nor HRP2 were correlated with EPO, indicating that *P. falciparum *infection did not affect the secretion of EPO (Table 2).

**Figure 3:**
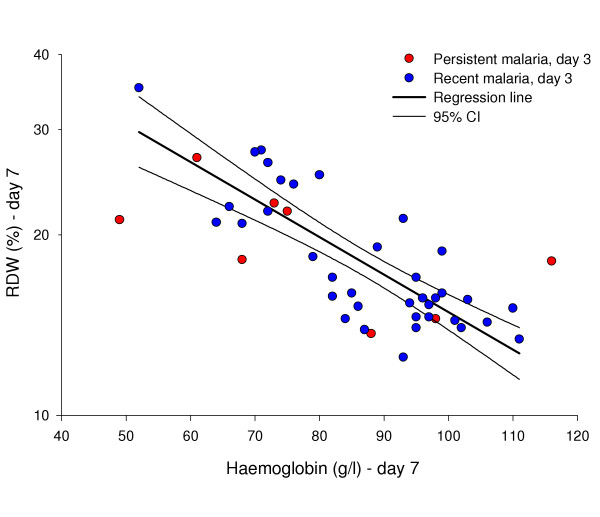
**Erythropoietic response to anaemia after clearance of *P. falciparum***. Association between haemoglobin and red cell distribution width (RDW) on day 7. Regression line and 95% confidence interval.

Forty patients in *group 2 *were followed up on day 7. In these, bone marrow suppression had receded and there was an inverse linear correlation between log RDW and Hb (R = -0.05, 95%CI: -0.06 – -0.04, P < 0.001, Figure 3). There was no difference between children who had a positive and those who had a negative test for pLDH on day three (P = 0.53), but two children remained severely anaemic.

### Cytokines

The plasma levels of TNF-α and IL-10 were comparable to those in normal Ghanaian children without symptoms of malaria or detectable parasitaemia [2]. There was an inverse linear correlation between log IL10 and Hb (R = -0.08, 95%CI: -0.14 – -0.03, P = 0.005). There was no correlation between log TNF-α and Hb (P = 0.89). The regression line between RDW and Hb was not influenced by TNFα, IL-10 or the IL-10/TNF-α ratio, irrespective of antigen test results (multiple regression analysis, IL-10, P = 0.40, TNF-α, P = 0.53, IL-10/TNF-α, P = 0.40). Analyses with sTfR as a dependent variable gave similar results (IL-10, P = 0.78, TNF-α, P = 0.35, IL-10/TNF-α, P = 0.63). On day 0 (i.e. prior to recruitment), patients in *group 2 *had elevated plasma IL-10 (mean (95% CI) 450.4 (291.5–609.3) pg/ml) and TNF-α (36.3 (25.7–51.3) pg/ml), but the levels were not correlated with Hb (IL-10, P = 0.31, TNF-α, P = 0.30, IL-10/TNF-α ratio, P = 0.21).

## Discussion

Children living in areas with endemic malaria are usually assumed to have malaria if they are hospitalized with severe anaemia that is preceded by an acute febrile illness. Such patients often have undetectable malaria parasites by microscopy of Giemsa-stained blood films, but their leukocytes commonly contain detectable malaria pigment [15]. It has previously been shown that the diagnosis may be aided by the use of rapid antigen detection tests, which can distinguish patients with malarial anaemia from those with anaemia from other causes [7], and this was supported in the present study. Thus, more than 90% of the patients with severe anaemia had detectable parasite antigens compared to only one third of those with normal or near normal Hb. Although microscopy is usually considered the gold standard for the diagnosis of malaria, these results suggest that the antigen detection methods, in particular those based on the detection of pLDH, may be superior to microscopy in the diagnosis of malarial anaemia.

In the present study, patients with detectable pLDH, a sign of persistent parasitaemia, were distinguished from those in whom only HRP2 could be detected as a sign of a cleared, recent infection. Only patients with persistent parasitaemia had signs of bone marrow suppression. This corresponds with previous investigations in which release of immature erythrocytes from the bone marrow coincided with the time of parasite clearance [1,6] and with findings in experimental malaria [16]. In one such study, subpatent *P. falciparum *infection in vaccinated Aotus monkeys led to severe bone marrow suppression that was rapidly reversed in response to mefloquine induced parasite clearance [17].

The association between *P. falciparum *infection and suppression of erythropoiesis has been debated. Menendez et al. found that levels of sTfR were increased in infants with malaria [18]. Similarly, Verhoef found increased levels of sTfR in children with malaria and concluded that there was no suppression of erythropoiesis [19]. However, in these studies it was not indicated if levels of sTfR were increased adequately compared to the degree of anaemia. Furthermore, increased levels of sTfR found in malaria patients might be caused by shedding of receptors from proliferating B-lymphocytes [16]. In the present study, however, there was no correlation between sTfR and lymphocyte count (data not shown). Due to the logistics of the study it was not possible to make reticulocyte counts but the finding that persistent, subpatent parasitaemia was associated with reduced levels of both RDW and sTfR strongly points toward parasite-induced suppression of erythropoiesis. This may explain the fact that healthy school children with so-called asymptomatic *P. falciparum *infection have reduced Hb and signs of bone marrow suppression [2]. Asymptomatic *P. falciparum *infections are usually accepted as a necessary evil in order to maintain immunity in individuals, who are at great and constant risk of malarial infections [20], and this approach is probably the only realistic option in the near future. However, data from the present study imply that the ultimate goal of malaria control ought to be complete parasite eradication due to the detrimental effects of persistent parasitaemia. This is consistent with a previous clinical trial that linked incomplete haematological recovery with lack of parasitological cure [21]. The Hb levels in infants living with high risk of malaria can be improved by impregnated bednets [22], malaria chemoprophylaxis [23] and presumptive intermittent treatment [24]. It is likely that this effect is mediated partly by a reduction in the risk of bone marrow suppression. From a clinical point of view this study suggests that in malarial endemic areas, antimalarial treatment should not be withheld from severely anaemic patients presenting with signs compatible with ongoing or recent malaria who have not been treated for malaria, even when parasites are undetectable by microscopy. Controlled clinical trials should be performed to determine whether patients who have already received a full course of antimalarial treatment would benefit from a repeat course, and in particular, if it is important to restrict this treatment to children with a positive pLDH test. Paediatric patients that return with repeated episodes of anaemia are common (ref. 7) and the management of these patients puts strain on the health system. Thus, the cost of improved diagnosis and management of these cases have the potential to benefit both the patients and the health system.

Several studies have shown that the suppressed bone marrow response to anaemia, which is a general feature of malaria, is not caused by insufficient secretion of EPO [1,25,26], but this is disputed by other investigations [27-29]. In agreement with the former view, similar levels of EPO in response to low Hb were found in patients with and without malaria. It has been suggested that the bone marrow suppression is a direct effect of TNF-α, which is elevated in malaria [30]. On the other hand, other investigations did not find that inflammatory cytokines to played a role in malarial dyserythropoiesis [31]. In addition, severe malarial anaemia is associated with relatively low TNF-α levels [32] and signs of systemic inflammation [33,34] (Awandare *et al. *unpublished data) compared with cerebral malaria and uncomplicated malaria. It is thus possible that the effect of cytokine perturbations in malarial anaemia is a lack of parasite control that leads to persistence of the infection and thus indirectly causes bone marrow suppression [32]. It has recently been proposed that phagocytosed haemozoin may play a role in the dyserythropoiesis of malaria through induction of 4-hydroxynonenal [35].

## Conclusion

This study has shown that the majority of severe anaemia cases in children living in areas with malaria transmission is due to malaria. This is also the case when no malaria parasites can be detected by microscopy, in which case antigen detection may lead to the diagnosis. These patients have suppressed erythropoiesis, which persists until the parasites have been cleared completely.

## Authors' contributions

Marie Helleberg and Jorgen A. L. Kurtzhals designed the study in collaboration with all co-authors. Marie Helleberg, Bamenla Q. Goka, George Obeng-Adjei and Onike Rodriques performed the clinical work; Marie Helleberg and Bartholomew D. Akanmori did the laboratory analysis. Marie Helleberg did the data analysis and drafted the manuscript. All authors contributed significantly to the final version of the manuscript. The authors do not have any commercial or other association that might pose a conflict of interest.

## Financial support

The study was supported by grants from the Danish Medical Research Council (SSVF grant no. 22-01-0343 and 22-03-0063) and the Danish International Development Assistance.
